# Health and Healthcare-Related Experiences Among Filipino University Students: A Cross-Sectional Descriptive and Unadjusted Comparative Study

**DOI:** 10.3390/nursrep16100345

**Published:** 2026-09-23

**Authors:** Melynn Grace Parcon, Sara Darias-Curvo, Cristo Manuel Marrero-González, Ángel Ramón Sabando-García

**Affiliations:** 1 Program in Medical and Pharmaceutical Sciences, Development and Quality of Life, University of La Laguna, 38200 Santa Cruz de Tenerife, Spain; 2 Centre for the Study of Social Inequality and Governance, University of La Laguna, 38200 Santa Cruz de Tenerife, Spain; sadacur@ull.edu.es; 3 Nursing Section, Department of Nursing, Faculty of Health Sciences, University of La Laguna, 38200 Santa Cruz de Tenerife, Spain; cmarrerg@ull.edu.es; 4 Department of Statistics, Pontifical Catholic University of Ecuador, Santo Domingo 230203, Ecuador

**Keywords:** university students, healthcare access, health insurance, social determinants of health, parental migration, mental health, preventive healthcare, health-seeking behavior, Philippines, nursing

## Abstract

**Background**: In the Philippines, despite recent health system reforms, barriers to healthcare access and financial protection persist, and the role of parental migration in shaping healthcare access among university students remains incompletely understood. Guided by the Social Determinants of Health framework and Andersen’s Behavioral Model of Health Services Use, this study aimed to describe health and healthcare-related experiences in a sample of university students in Cavite; compare self-reported mental-health-related experiences by current parental overseas employment; and explore bivariate associations between selected student characteristics and self-reported health-insurance status. **Methods**: A cross-sectional survey of 485 university students aged 18–25 was conducted between June and July 2023 in Cavite, Philippines. Conventional statistical analyses were used to describe the sample and assess unadjusted associations between selected variables. **Results**: Most students reported no health insurance (74.2%), and preventive healthcare engagement was low. 11.3% had parental migration experience. No consistent overall pattern of statistically significant differences was observed between students with and without parental migration experience across the self-reported mental-health-related experiences examined. **Conclusions**: Self-reported mental-health-related experiences were common among participating students. Although no consistent overall differences were identified according to parental migration experience, the findings suggest that self-reported mental-health-related experiences may be shaped by interacting academic, socioeconomic, and familial factors. Low perceived insurance coverage and limited preventive healthcare engagement highlight persistent barriers to healthcare access. Strengthening university-based mental health services, health literacy initiatives, and preventive healthcare engagement remains essential.

## 1. Introduction

Safeguarding the health of university students is a public health priority because the transition to higher education coincides with the peak period of risk for onset of mental illness and exposure to multiple stressors [1,2]. The university student population constitutes a substantial share of emerging adults, and higher education itself depends on good mental health while serving as a major determinant of individual and societal development [3,4]. During this transition, students face numerous life events and must continuously rebuild support networks, negotiate stigma, and decide when and how to seek help, underscoring the importance of accessible and acceptable health services [5]. Ensuring that university students can effectively access healthcare is therefore essential to protecting their well-being and their ability to benefit from higher education [6,7].

In the Philippines, healthcare access is shaped by recent policy reforms and persistent structural barriers [8,9]. The Mental Health Act of 2018 and the Universal Health Care Act of 2019 were enacted to expand equitable access, including through PhilHealth insurance coverage for mental health services [10]. The policy environment has also changed since data collection. PhilHealth Circular No. 2023-0018 introduced an outpatient mental health benefits package after the survey period, and PhilHealth currently lists outpatient mental health services among its benefits. Data reported here therefore describe a coverage environment that predates this expansion, and current entitlements should not be inferred from respondents’ 2023 answers [11]. Empirical data from medically underserved Filipino communities further show that most adults are uninsured, have low incomes, and rely primarily on family and friends for health information, with around one-third not seeking healthcare professionals even when ill [12,13]. These patterns suggest that, despite universal health coverage reforms, significant barriers to equitable healthcare access and financial protection remain relevant to young adults, including university students. Limited knowledge of available health services and other aspects of health literacy may further constrain healthcare utilization by reducing individuals’ ability to navigate healthcare and insurance systems [14].

Mental health and health-related behaviors are central to understanding how students interact with health services. Emerging adulthood is a peak period for mental health problems, and the transition to university heightens both risks and opportunities for intervention, yet the evidence base remains limited and varied [15]. Among Filipinos, help-seeking for mental health problems is often delayed, with a general reluctance toward formal services, preference for close family and friends, and use of specialist care only when problems become severe [10]. More broadly, self-medication, prayer, and informal providers are common responses to illness in medically underserved Filipino communities, indicating that lifestyle and coping behaviors are closely linked to patterns of healthcare utilization [12,13]. For university students, these health-related behaviors may shape both the timing and likelihood of engaging with preventive, curative, and mental healthcare services, thereby influencing broader patterns of healthcare utilization [16].

Parental labor migration is a pervasive feature of Filipino society and shapes the social context in which many young people grow up [17]. Overseas Filipino Workers contribute substantially to family socioeconomic well-being, yet migration may also alter family dynamics, caregiving arrangements, and social support during critical developmental periods [18,19]. Previous studies have reported associations between parental migration and adverse psychological outcomes among left-behind children, although findings from the Philippines and other Southeast Asian settings remain heterogeneous and highly context-dependent [20,21,22]. Accordingly, parental migration is considered in the present study as one contextual social determinant that may influence health behaviors and healthcare access among university students, rather than as the sole or primary determinant of these outcomes. While examining parental migration provides insight into the influence of a specific contextual social determinant on student health, healthcare access is inherently shaped by the interaction of multiple predisposing, enabling, and need factors [23].

Despite policy efforts to expand healthcare coverage, basic descriptive evidence on the health profile, insurance status and healthcare-seeking patterns of Filipino university students remains limited. In addition, there is limited evidence on whether self-reported mental-health-related experiences differ by current parental overseas employment within a university setting. This study therefore provides descriptive and unadjusted comparative evidence rather than testing causal pathways or complex interactions among determinants.

Accordingly, this study investigated the health profile and healthcare access of Filipino university students, drawing primarily on the Social Determinants of Health Framework while using Andersen’s Behavioral Model to provide additional perspectives on healthcare access. Within Andersen’s Behavioral Model, health insurance was conceptualized as a key enabling resource that facilitates healthcare access and financial protection and was therefore used as a proxy indicator of one dimension of healthcare access within this study. This study aimed to describe the health profile and healthcare-related experiences of Filipino university students, compare self-reported mental-health-related experiences by current parental overseas employment, and explore unadjusted associations between selected student characteristics and self-reported health-insurance status.

## 2. Materials and Methods

### 2.1. Study Design and Setting

This study utilized a cross-sectional survey design conducted between 22 June and 19 July 2023. A cross-sectional design was selected to provide an initial assessment of health, healthcare access, health-related behaviors, and parental migration within a defined period. This design cannot establish temporal sequence or causality. The setting was Cavite State University–Imus Campus, located in Cavite, Philippines. The university was selected as the study setting because it provided access to the target population and served students from diverse socioeconomic backgrounds, making it a suitable setting for an initial exploratory investigation of health experiences, lifestyle behaviors, family contexts, and healthcare-related experiences.

### 2.2. Participants

The study included Filipino university students aged 18–25 years enrolled at Cavite State University–Imus Campus. The eligibility criteria were being a Filipino student, being 18–25 years of age and being enrolled at the study campus during the data-collection period. Because the study used convenience sampling, participants were recruited from selected classes whose faculty members agreed to facilitate distribution of the survey. Recruitment was therefore concentrated in a single academic unit.

### 2.3. Measures

Data were collected using a structured self-administered questionnaire. The variables analyzed in this study included sociodemographic characteristics (age, sex, birthplace, religion, employment status, and parents’ educational attainment); parental migration experience (whether at least one parent was currently working abroad); healthcare-related variables (health insurance status, type of insurance, self-rated health, knowledge of available health services, and frequency of health check-ups); lifestyle behaviors (smoking, alcohol consumption, prohibited substance use, exercise frequency, and sleep duration); and mental-health-related experiences during the previous three months, including emotional experiences, interference with work and academic activities, prolonged low mood, interpersonal relationships, sleep quality, and self-reported mental health concerns. Questionnaire items used dichotomous, categorical, and ordinal response formats according to the variable being assessed.

### 2.4. Instrument Development

The research team developed the questionnaire specifically for this study; items were not copied from a standardized diagnostic or health-literacy instrument. The analytic questionnaire comprised 42 closed-response items covering demographics, self-rated health, self-reported insurance, knowledge of community health services, medical check-ups, health behaviors, current parental overseas employment, and mental-health-related experiences. Eight optional open-ended questions concerning parental overseas employment were present in the survey file but were not analyzed in this quantitative report.

Three licensed psychometricians reviewed wording, clarity, content relevance, appropriateness, and sensitivity. The revised questionnaire was pilot tested with 53 students for comprehensibility and contextual relevance. These procedures support face and content relevance but do not establish psychometric validity, diagnostic accuracy, or reliability of multi-item scales. The mental health items are therefore reported as self-reported experiences and must not be interpreted as diagnosed disorders or validated screening results. Self-reported knowledge of services is not treated as a validated measure of health literacy.

### 2.5. Data Collection

Data were collected from 22 June to 19 July 2023 through classroom visits and official class group chats in selected classes whose faculty members agreed to facilitate distribution. Students accessed an anonymous online questionnaire through a QR code or link. Participation was voluntary. The number of students invited and the campus enrolment denominator available to the research team were not recorded; consequently, a response rate could not be calculated.

The raw file contained 487 submissions. One record contained only a timestamp and no questionnaire responses, and one respondent selected the age category “30 years or older”; both were excluded. The final analytic sample was 485. Because recruitment was non-probabilistic and limited to selected classes at one campus, the sample should not be regarded as representative of the campus or of Filipino university students generally.

### 2.6. Statistical Analysis

Data were analyzed using IBM SPSS Statistics version 25 (IBM Corp., Armonk, NY, USA), Minitab version 18(Minitab, LLC, State College, PA, USA) and Python-based tools within Orange Data Mining version 3.39.0 (University of Ljubljana, Ljubljana, Slovenia). Descriptive statistics were used to summarize participants’ characteristics. Comparisons of self-reported mental-health-related experiences between students with and without parental migration experience were assessed using one global test per variable. Pearson’s chi-square test was used when expected-cell assumptions were adequate, while fixed-margin Monte Carlo exact tests were used for sparse contingency tables. Category-specific percentage-point differences between students with and without parental migration experience were reported descriptively with 95% confidence intervals. Cramér’s V was used to report effect sizes for the global associations. Logistic regression was not performed because the analysis was designed as an unadjusted group comparison, the parental-migration subgroup was relatively small (*n* = 55), and key potential confounders were not sufficiently measured to support a defensible, adjusted model. Benjamini–Hochberg false-discovery-rate adjustment was applied to account for multiple comparisons, separately across the six parental-migration comparisons and the four exploratory insurance-status comparisons. The participant recruitment and study flow are summarized in Figure 1.

## 3. Results

### 3.1. Sociodemographic Profile

The analytic sample comprised 485 students; 385 (79.4%) were aged 18–21 years, 351 (72.4%) were women, and 55 (11.3%) reported at least one parent currently working abroad. Complete distributions are shown in Table 1.

### 3.2. Health Access and Health Condition

Most respondents rated their recent health as good or excellent (291/485, 60.0%). Nevertheless, 360 (74.2%) reported that they did not have health insurance, and 342 (70.5%) reported obtaining a medical check-up only when needed. Because legal entitlement was not verified, the insurance result reflects respondents’ perceived or reported status.

Self-reported health and healthcare-related characteristics are presented in Table 2.

### 3.3. Health-Related Behaviors

Most respondents reported not smoking (96.3%) and not using prohibited substances (97.3%). Exercise was occasional for 232 (47.8%), and 129 (26.6%) reported no exercise. The survey contained both a general alcohol-use item and a frequency item; the latter was used in the exploratory insurance comparison.

Self-reported health-related behaviors are summarized in Table 3.

### 3.4. Self-Reported Mental-Health-Related Experiences

Among respondents who reported health problems or mental-health-related experiences, 330 (68.0%) indicated that depression-, sadness-, or anxiety-related feelings interfered with work at least sometimes, 363 (74.8%) reported that mental health concerns interfered with academic tasks at least sometimes, and 382 (78.8%) reported sleeping fewer than seven hours. These findings represent item-level self-reports and should not be interpreted as clinical prevalence estimates.

Self-reported mental-health-related experiences are presented in Table 4.

### 3.5. Comparison Between Students with and Without Parental Migration Experience

Fifty-five students (11.3%) reported parental migration experience. None of the six overall self-reported mental-health-related response distributions showed a statistically significant difference between students with and without parental migration experience after false-discovery-rate adjustment. Descriptive category-level differences were observed, but these should not be interpreted as inferential findings.

Comparisons by current parental overseas employment are presented in Table 5.

### 3.6. Exploratory Associations with Self-Reported Insurance Status

Table 6 presents the distributions for four prespecified exploratory comparisons. After adjustment across these four tests, academic interference, alcohol-consumption frequency, and prohibited-substance use were statistically flagged, with small effect sizes. The alcohol and prohibited-substance tables contained very small cells; these associations are unstable and should not be interpreted as predictive or causal. The medical-check-up distribution was not statistically associated with reported insurance status after adjustment. These exploratory comparisons were unadjusted for confounding and cannot establish independent associations, temporal relationships, or causality. Sparse categories are shown rather than collapsed to preserve the recorded response options; results involving sparse cells require caution. Only three insured respondents reported prohibited-substance use and none of the uninsured respondents reported use.

## 4. Discussion

The present study examined the health profile, mental health and healthcare access of participating university students at Cavite State University–Imus Campus, whose sociodemographic characteristics are presented in Table 1, within the complementary frameworks of the Social Determinants of Health and Andersen’s Behavioral Model of Health Services Use. Overall, the findings indicate that self-reported mental health concerns, lifestyle behaviors, and healthcare access challenges are common among the study population, reflecting the multidimensional nature of health in this population. No consistent overall pattern of statistically significant differences in self-reported mental-health-related experiences was observed between students with and without parental migration experience, suggesting that parental migration alone may not be sufficiently associated with self-reported mental-health-related experiences within this cohort. At the same time, the low perceived insurance coverage and patterns of healthcare engagement highlight reported limitations in healthcare-related financial protection and healthcare access.

### 4.1. Overview of the Findings and Conceptual Interpretation

The findings of this study are best understood through the complementary perspectives of the Social Determinants of Health (SDOH) framework and Andersen’s Behavioral Model of Health Services Use. The SDOH framework emphasizes that health and healthcare access are shaped by broader social, economic, and environmental conditions rather than individual characteristics alone [24], whereas Andersen’s model provides an operational explanation of how predisposing, enabling, and need factors are associated with healthcare utilization [23]. Within this conceptual framework, parental migration represents a contextual social determinant that may shape family environments and social support, while health insurance functions as an enabling resource facilitating healthcare access and financial protection, and mental health reflects an important need factor influencing perceived healthcare requirements and help-seeking behavior. Lifestyle and health-seeking behaviors, including preventive care practices and healthcare utilization patterns, further reflect the interaction between individual behaviors and the broader social context.

### 4.2. Parental Migration as Contextual Social Determinant

While parental migration was conceptualized in this study as a contextual social determinant within the students’ health environments, none of the six overall response distributions of self-reported mental-health-related experiences differed statistically significantly between students with and without parental migration experience after false-discovery-rate adjustment. Descriptive category-level differences were observed (Table 5). Given the relatively small parental-migration subgroup (*n* = 55, 11.3%), the comparative analyses may have had limited power to detect modest associations. Therefore, these findings should not be interpreted as evidence that parental migration has no association with these self-reported mental-health-related experiences, but rather that its associations may be complex, context-dependent, and related to other social, structural, and familial factors. Previous studies from low-and middle-income countries have reported associations between parental migration and poorer mental-health-related outcomes among left-behind children and adolescents, including depression, anxiety, behavioral problems, and substance use [21,25,26]. However, evidence from the Philippines and other Southeast Asian settings suggests that these associations are highly context-dependent and may be moderated by caregiving arrangements, family cohesion, remittance-related financial support, and sustained communication with migrant parents [20,27,28].

Consistent with the Social Determinants of Health framework, parental migration should be viewed as one contextual determinant operating alongside economic resources, family support systems, educational demands, and access to health-promoting resources [22,24]. In the present study, mental health concerns were prevalent across both groups (Table 5), suggesting that self-reported mental-health-related experiences may also be associated with broader structural and academic factors, rather than parental migration status alone, consistent with a multidimensional perspective in which parental migration represents one component of a broader network of interacting factors associated with university students’ health [22,29,30].

### 4.3. Mental Health Concerns Among University Students

Broader socioeconomic conditions may also shape self-reported mental-health-related experiences across both groups. As the study was conducted in a public university serving students from diverse but often financially constrained backgrounds, students without parental migration experience may likewise experience economic and academic stressors. Some migrant-parent households may also receive remittance-related financial support, which has been reported to mitigate potential adverse associations between parental migration and family well-being [22,30]. However, because remittance dependence and household socioeconomic status were not directly assessed in the present study, this interpretation remains speculative and should be interpreted cautiously.

Likewise, self-reported mental health challenges among Filipino students should not be interpreted solely as individual-level concerns, but as manifestations of broader structural vulnerabilities shaped by educational, economic, and health-system conditions [24,31]. This perspective is especially relevant for students from transnational family contexts, where changing caregiving arrangements, emotional distance, and unequal access to familial and institutional support may characterize students’ experiences [20,22,28,32]. However, self-reported mental-health-related experiences were prevalent across both groups (Table 5). Emotional symptoms, sleep problems, and academic interference were common, echoing broader concerns about student well-being nationwide [33,34]. These results suggest that self-reported mental health challenges among youth may also be associated with academic pressures, lifestyle factors, and environmental stressors (Table 3) rather than parental migration alone, a trend also noted in studies of Filipino university students’ stress and coping [29].

### 4.4. Healthcare Access and Health Insurance

The findings related to healthcare access highlight important barriers to healthcare utilization among the sample. Most participants reported no health insurance, two-thirds reported no or only partial knowledge of available health services (67.4%, Table 2), and the majority sought medical care only when needed rather than engaging in routine preventive check-ups. These patterns are consistent with previous studies reporting barriers to healthcare utilization among Filipino adolescents and young adults, including limited awareness of available services and low engagement with preventive care [35,36]. They also align with national evidence indicating that out-of-pocket healthcare costs continue to discourage preventive healthcare utilization in the Philippines [37].

Although the Philippine Universal Health Care Act of 2019 legally provides PhilHealth coverage to all Filipino citizens, legal entitlement does not necessarily translate into awareness or utilization of health insurance benefits [38,39]. Therefore, the high proportion of respondents reporting no health insurance may reflect perceived insurance status or limited awareness of PhilHealth coverage, in addition to actual lack of insurance, which is consistent with the observed gaps in knowledge regarding available healthcare services.

Interestingly, a similar pattern of limited preventive healthcare utilization and restricted knowledge of available health services was observed in previous studies involving Filipino migrants, despite the fact that the vast majority of participants had access to health insurance [40,41]. This consistency across different healthcare systems suggests that healthcare-seeking behaviors observed among young adults may not be fully explained by the availability of health services or self-reported insurance coverage alone [42,43]. This observation further supports Andersen’s proposition that enabling resources alone may not fully determine healthcare utilization when predisposing beliefs and behavioral patterns remain unchanged. Although this interpretation remains tentative and warrants longitudinal investigation, it highlights the potential relevance of enduring behavioral and cultural factors on healthcare utilization. In the present study, insurance status was associated, in unadjusted exploratory comparisons, with academic interference, alcohol-consumption frequency, and prohibited-substance use (Table 6). These associations were small and should be interpreted cautiously, particularly given the sparse categories in the alcohol and prohibited-substance responses. These unadjusted associations cannot establish independent associations, temporal relationships, or causality. In the substance-use items, only three insured respondents reported prohibited-substance use and none of the uninsured respondents reported use, so this association should be interpreted with particular caution. Within Andersen’s Behavioral Model of Health Services Use, health insurance represents an important enabling resource that facilitates access to healthcare services and reduces financial barriers to care. However, the persistence of low preventive healthcare engagement even in settings with insurance coverage suggests that enabling resources alone may be insufficient to promote healthcare utilization. Predisposing characteristics such as health beliefs, together with enabling and need factors, may also play important roles in shaping health service use [23,44,45]. Furthermore, although health literacy was not directly measured in the present study, participants’ limited self-reported knowledge of available health services may reflect challenges in healthcare system navigation that could reduce timely utilization of preventive and curative services. These findings reinforce the importance of strengthening both structural enabling resources and behavioral interventions to improve healthcare access among university students.

### 4.5. Implications for Policy and Practice

The present findings have important implications for university health promotion and public health policy. Given the interrelated factors influencing healthcare access among university students, interventions should extend beyond mental health services alone to include initiatives to improve knowledge of available health services and healthcare-system navigation, promotion of preventive healthcare behaviors, and strategies to improve awareness and enrollment in health insurance programs such as PhilHealth. Universities may serve as important settings for integrated health promotion by facilitating early healthcare engagement, strengthening referral pathways to health services, and providing accessible information on available healthcare resources and financial protection mechanisms [7]. Such holistic interventions may contribute to reducing barriers to healthcare access and promoting equitable health outcomes among university students, particularly in resource-constrained settings.

This study has several limitations. The cross-sectional design provides a snapshot of the study population and permits assessment of associations but does not establish temporal relationships or causal effects between parental migration, health-related factors, and healthcare access. The use of convenience sampling from selected classes may have introduced selection bias and limits the generalizability of the findings. Because recruitment was conducted through participating classes using convenience sampling, the exact number of students invited to participate was not recorded during data collection; therefore, a response rate could not be determined. Students who participated may also differ systematically from those who were not reached or did not participate, which may limit the representativeness of the sample. Selection bias cannot be excluded because recruitment was conducted through participating classes rather than through a sampling frame covering all eligible students. Recruitment was therefore concentrated in a single academic unit. The direction and magnitude of any resulting bias could not be assessed because the number and characteristics of students who were invited but did not participate were not recorded. As the study was conducted at a single university campus, institutional, geographic, and socioeconomic variation across other Philippine universities was not captured. The relatively small subgroup of participants with parental migration experience may have reduced the statistical power of comparative analyses. The parental-migration measure was also limited to whether at least one parent was currently working abroad and did not capture duration of separation, which parent migrated, remittance stability, caregiving arrangements, communication, or previous migration-related experiences. Additionally, the use of self-reported measures may be subject to reporting bias, particularly in the context of self-reported mental-health-related experiences, where stigma may influence responses. The mental-health-related items were developed for this study and were not derived from a validated diagnostic or screening instrument; therefore, the reported findings represent self-reported mental-health-related experiences and should not be interpreted as clinical diagnoses. Another important limitation is that household socioeconomic status, parental income, and remittance dependence were not directly measured. These variables represent key structural and enabling resources within the Social Determinants of Health framework and Andersen’s Behavioral Model and may be associated with healthcare access and health-related behaviors. Their omission limits the ability to fully examine the broader socioeconomic mechanisms underlying the observed associations and should be addressed in future research. Other potential confounders, including financial strain, transport, service supply, and caregiving arrangements, were not measured sufficiently to support adjusted modelling. Health insurance represents one financial enabling resource within healthcare access, but it does not capture healthcare access comprehensively. Some response categories were sparse, and the four insurance comparisons remain exploratory even after false-discovery rate adjustment. However, healthcare access is inherently multifactorial and cannot be fully captured by insurance status alone, as it also involves service availability, affordability, awareness, and actual healthcare utilization. Future research using larger and more representative samples, as well as more comprehensive and validated tools, is recommended. Despite these limitations, this study provides important evidence on the health and healthcare access of Filipino university students and offers practical directions for strengthening university health promotion initiatives.

Based on the findings of this study, universities should strengthen mental health services by expanding counseling programs and developing accessible and student-centered support systems. Promoting knowledge of available health services and strengthening healthcare system navigation skills are also essential to encourage appropriate help-seeking behaviors and preventive healthcare engagement. Although health literacy was not directly assessed in this study, participants’ limited knowledge of available health services may reflect broader challenges in accessing, understanding, and navigating the healthcare system. Initiatives to improve knowledge of available health services and healthcare-system navigation should likewise be incorporated into university health promotion programs to empower students to access, understand, and effectively use health information and services. Universities may also consider providing context-sensitive support for students with parental migration experience, recognizing the diverse emotional, familial, and socioeconomic experiences that may be present among these students. Finally, stronger partnerships between universities and local health units may help improve access to preventive and responsive healthcare services for the student population. Future interventions should strengthen knowledge of available health services, mental health promotion, preventive healthcare engagement, and healthy lifestyle behaviors among university students through multidisciplinary and nursing-led initiatives.

## 5. Conclusions

Among 485 participating students at one public university campus in Cavite, self-reported mental-health-related difficulties, limited routine check-ups, and low perceived insurance coverage were common. The study found no evidence of overall differences in the measured mental-health-related response distributions by current parental overseas employment, but the estimate was imprecise. Associations with self-reported insurance status were small, unadjusted, and exploratory. The results support accessible student mental health services, preventive-care information, and assistance with PhilHealth navigation while underscoring the need for multisite probability-based or longitudinal research using validated measures and verified coverage information.

## Figures and Tables

**Figure 1:**
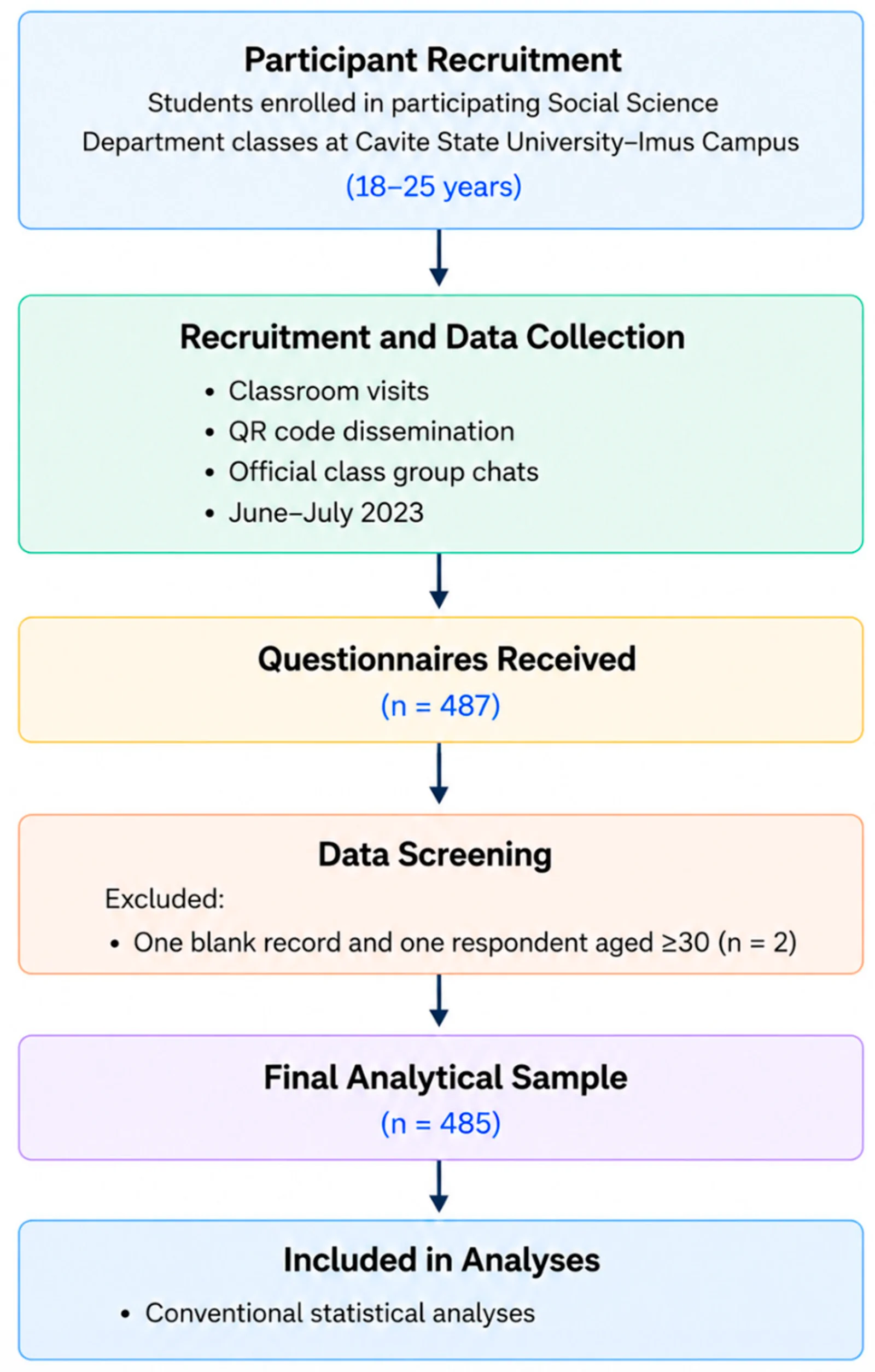
Participant recruitment and study flow.

**Table 1:** Sociodemographic characteristics of the analytic sample (N = 485).

Variable	Category	*n*	%
Age	18–21 years	385	79.4
	22–25 years	100	20.6
Sex	Male	134	27.6
	Female	351	72.4
Birth region	Luzon	457	94.2
	Visayas	16	3.3
	Other	12	2.5
Mother’s education	No formal education	1	0.2
	Elementary	27	5.6
	High school	179	36.9
	Vocational	23	4.7
	College	207	42.7
	Postgraduate	19	3.9
	Prefer not to say	29	6.0
Father’s education	No formal education	3	0.6
	Elementary	37	7.6
	High school	161	33.2
	Vocational	41	8.5
	College	184	37.9
	Postgraduate	18	3.7
	Prefer not to say	41	8.5
Religion	Catholic	325	67.0
	Other Christian denomination	143	29.5
	Other religion	17	3.5
Civil status	Single	482	99.4
	Married	3	0.6
Currently working	No	428	88.2
	Yes	57	11.8
Parent currently working abroad	No	430	88.7
	Yes	55	11.3

**Table 2:** Self-reported health and healthcare-related characteristics (N = 485).

Variable	Category	*n*	%
Self-reported health insurance	No	360	74.2
	Yes	125	25.8
Health in previous 3 months	Very poor	6	1.2
	Poor	41	8.5
	Fair	147	30.3
	Good	242	49.9
	Excellent	49	10.1
Knowledge of community health services	No	124	25.6
	Some services	203	41.9
	Yes	158	32.6
Medical check-ups	Does not attend	79	16.3
	Only when needed	342	70.5
	Once a year	38	7.8
	Once every 6 months	17	3.5
	Once every 3 months	9	1.9
Insurance type among valid follow-up answers	Public/PhilHealth	91	74.6 *
	Private	20	16.4 *
	Both public and private	11	9.0 *

* Insurance-type percentages use 122 respondents who answered “yes” to insurance and supplied a valid type. Three respondents who answered “yes” selected “not applicable”; one respondent who answered “no” selected a public type. These inconsistencies were retained as reported and are disclosed here.

**Table 3:** Self-reported health-related behaviors (N = 485).

Variable	Category	*n*	%
Smoking	No	467	96.3
	Yes	18	3.7
General alcohol-use response	Never	149	30.7
	Rarely	160	33.0
	Sometimes	142	29.3
	Often	28	5.8
	Always	6	1.2
Alcohol-consumption frequency	Never	150	30.9
	Occasionally	317	65.4
	Once a week	10	2.1
	Twice a week	2	0.4
	Three times a week or more	6	1.2
Prohibited-substance use	No	472	97.3
	Yes	3	0.6
	Prefer not to say	10	2.1
Exercise (≥30 min)	None	129	26.6
	Occasionally	232	47.8
	3–5 times/week	105	21.6
	Every day	19	3.9

Note: Only three respondents reported prohibited-substance use; estimates involving this category should be interpreted cautiously.

**Table 4:** Self-reported mental-health-related experiences (N = 485).

Variable	Category	*n*	%
Feelings interfered with work	Never	48	9.9
	Rarely	107	22.1
	Sometimes	180	37.1
	Often	104	21.4
	Always	46	9.5
Mental health concerns interfered with academic tasks	Never	36	7.4
	Rarely	86	17.7
	Sometimes	185	38.1
	Often	117	24.1
	Always	61	12.6
Low mood/energy > 2 consecutive weeks	No	73	15.1
	Yes	200	41.2
	Sometimes	212	43.7
Experienced a mental-health-related problem	No	139	28.7
	Yes	285	58.8
	Prefer not to say	61	12.6
Negative effect on relationships	Never	65	13.4
	Rarely	102	21.0
	Sometimes	205	42.3
	Often	92	19.0
	Always	21	4.3
Average sleep quality	Very poor	56	11.5
	Poor	141	29.1
	Fair	186	38.4
	Good	91	18.8
	Excellent	11	2.3
Average sleep duration	<4 h	67	13.8
	4–6 h	315	64.9
	7–9 h	100	20.6
	>10 h	3	0.6

**Table 5:** Mental-health-related experiences by current parental overseas employment.

Variable	Category	Parent Abroad *n* = 55, *n* (%)	No Parent Abroad *n* = 430, *n* (%)	Difference, pp [95% CI]	Global Test
Self-reported mental health-related problem	No	16 (29.1)	123 (28.6)	+0.5 [−14.3, +17.6]	χ^2^ = 0.90; *p* = 0.638; q = 0.718; V = 0.043
	Yes	30 (54.5)	255 (59.3)	−4.8 [−22.3, +12.4]	
	Prefer not to say	9 (16.4)	52 (12.1)	+4.3 [−6.7, +18.9]	
Effect on relationships	Never	7 (12.7)	58 (13.5)	−0.8 [−10.7, +13.4]	MC = 7.13; *p* = 0.125; q = 0.543; V = 0.121
	Rarely	9 (16.4)	93 (21.6)	−5.3 [−16.9, +10.3]	
	Sometimes	28 (50.9)	177 (41.2)	+9.7 [−7.8, +27.0]	
	Often	6 (10.9)	86 (20.0)	−9.1 [−18.9, +5.3]	
	Always	5 (9.1)	16 (3.7)	+5.4 [−2.0, +17.3]	
Sleep quality	Very poor	9 (16.4)	47 (10.9)	+5.4 [−5.4, +19.9]	MC = 6.16; *p* = 0.181; q = 0.543; V = 0.113
	Poor	10 (18.2)	131 (30.5)	−12.3 [−24.8, +4.0]	
	Fair	23 (41.8)	163 (37.9)	+3.9 [−12.8, +21.5]	
	Good	13 (23.6)	78 (18.1)	+5.5 [−7.7, +21.6]	
	Excellent	0 (0.0)	11 (2.6)	−2.6 [−4.5, +5.1]	
Sleep duration	less than 4 h	10 (18.2)	57 (13.3)	+4.9 [−6.6, +20.0]	MC = 1.44; *p* = 0.718; q = 0.718; V = 0.054
	4 to 6 h	35 (63.6)	280 (65.1)	−1.5 [−19.0, +14.6]	
	7 to 9 h	10 (18.2)	90 (20.9)	−2.7 [−14.8, +13.0]	
	more than 10 h	0 (0.0)	3 (0.7)	−0.7 [−2.0, +6.3]	
Interference with work	Never	5 (9.1)	43 (10.0)	−0.9 [−9.3, +12.1]	χ^2^ = 2.61; *p* = 0.625; q = 0.718; V = 0.073
	Rarely	10 (18.2)	97 (22.6)	−4.4 [−16.6, +11.5]	
	Sometimes	24 (43.6)	156 (36.3)	+7.4 [−9.6, +24.9]	
	Often	9 (16.4)	95 (22.1)	−5.7 [−17.4, +9.8]	
	Always	7 (12.7)	39 (9.1)	+3.7 [−5.9, +17.3]	
Interference with academic tasks	Never	6 (10.9)	30 (7.0)	+3.9 [−4.7, +16.9]	MC = 3.79; *p* = 0.438; q = 0.718; V = 0.088
	Rarely	6 (10.9)	80 (18.6)	−7.7 [−17.5, +6.6]	
	Sometimes	25 (45.5)	160 (37.2)	+8.2 [−8.8, +25.7]	
	Often	12 (21.8)	105 (24.4)	−2.6 [−15.7, +13.8]	
	Always	6 (10.9)	55 (12.8)	−1.9 [−11.2, +11.9]	

Notes: Difference = percentage in the “parent abroad” group minus percentage in the “no parent abroad” group. CI = Newcombe–Wilson confidence interval; pp = percentage points; q = Benjamini–Hochberg-adjusted *p* value across six global tests; V = Cramér’s V; MC = fixed-margin Monte Carlo exact test with 200,000 samples. Pearson χ^2^ was used when expected-count assumptions were adequate.

**Table 6:** Selected variables by self-reported health-insurance status.

Variable	Category	Reported Insured *n* = 125, *n* (%)	Reported Uninsured *n* = 360, *n* (%)	Global Test
Interference with academic tasks	Never	16 (12.8)	20 (5.6)	χ^2^ = 10.75; *p* = 0.030; q = 0.039; V = 0.149
	Rarely	23 (18.4)	63 (17.5)	
	Sometimes	37 (29.6)	148 (41.1)	
	Often	30 (24.0)	87 (24.2)	
	Always	19 (15.2)	42 (11.7)	
Frequency of medical check-ups	I don′t go to medical check-ups	14 (11.2)	65 (18.1)	MC = 7.86; *p* = 0.094; q = 0.094; V = 0.127
	Only when needed	87 (69.6)	255 (70.8)	
	Once a year	13 (10.4)	25 (6.9)	
	Once in 6 months	7 (5.6)	10 (2.8)	
	Once in 3 months	4 (3.2)	5 (1.4)	
Alcohol-consumption frequency	Never	39 (31.2)	111 (30.8)	MC = 16.78; *p* = 0.002; q = 0.008; V = 0.186
	Occasionally	76 (60.8)	241 (66.9)	
	Once a week	8 (6.4)	2 (0.6)	
	Twice a week	0 (0.0)	2 (0.6)	
	Thrice a week or more	2 (1.6)	4 (1.1)	
Use of prohibited substances	No	118 (94.4)	354 (98.3)	MC = 9.85; *p* = 0.011; q = 0.022; V = 0.142
	Yes	3 (2.4)	0 (0.0)	
	Prefer not to say	4 (3.2)	6 (1.7)	

Notes: q = Benjamini–Hochberg-adjusted *p* value across four exploratory global tests; V = Cramér’s V; MC = fixed-margin Monte Carlo exact test with 200,000 samples. Sparse categories are shown rather than collapsed to preserve the recorded response options; results involving sparse cells require particular caution.

## Data Availability

The data presented in this study are available from the corresponding author upon reasonable request. The data are not publicly available because they contain information that could compromise the privacy and confidentiality of the study participants.

## Public Involvement Statement

No public involvement in any aspect of this research.

## Guidelines and Standards Statement

This manuscript was prepared in accordance with the Strengthening the Reporting of Observational Studies in Epidemiology (STROBE) Statement for cross-sectional studies.
